# L-Asparaginase from *Penicillium sizovae* Produced by a Recombinant *Komagataella phaffii* Strain

**DOI:** 10.3390/ph15060746

**Published:** 2022-06-14

**Authors:** Marcela Freitas, Paula Souza, Mauricio Homem-de-Mello, Yris M. Fonseca-Bazzo, Damaris Silveira, Edivaldo X. Ferreira Filho, Adalberto Pessoa Junior, Dipak Sarker, David Timson, João Inácio, Pérola O. Magalhães

**Affiliations:** 1 Health Sciences School, University of Brasilia, Brasilia 70910-900, Brazil; marcelamdf1@gmail.com (M.F.); paulasouza@unb.br (P.S.); mauriciohmello@unb.br (M.H.-d.-M.); yrisfonseca@unb.br (Y.M.F.-B.); damaris@unb.br (D.S.); 2 Institute of Biological Sciences, University of Brasilia, Brasilia 70910-900, Brazil; eximenes@unb.br; 3 Department of Biochemical and Pharmaceutical Technology, University of Sao Paulo, Sao Paulo 05508-000, Brazil; pessoajr@usp.br; 4 School of Pharmacy and Biomolecular Sciences, University of Brighton, Brighton BN2 4GJ, UK; d.k.sarker@brighton.ac.uk (D.S.); d.timson@brighton.ac.uk (D.T.); j.inacio@brighton.ac.uk (J.I.)

**Keywords:** L-asparaginase, acute lymphoblastic leukemia, cloning, expression, *Penicillium sizovae*, *Komagataella phaffii*

## Abstract

L-asparaginase is an important enzyme in the pharmaceutical field used as treatment for acute lymphoblastic leukemia due to its ability to hydrolyze L-asparagine, an essential amino acid synthesized by normal cells, but not by neoplastic cells. Adverse effects of L-asparaginase formulations are associated with its glutaminase activity and bacterial origin; therefore, it is important to find new sources of L-asparaginase produced by eukaryotic microorganisms with low glutaminase activity. This work aimed to identify the L-asparaginase gene sequence from *Penicillium sizovae*, a filamentous fungus isolated from the Brazilian Savanna (Cerrado) soil with low glutaminase activity, and to biosynthesize higher yields of this enzyme in the yeast *Komagataella phaffii*. The L-asparaginase gene sequence of *P. sizovae* was identified by homology to L-asparaginases from species of *Penicillium* of the section *Citrina*: *P. citrinum* and *P. steckii*. Partial L-asparaginase from *P. sizovae*, lacking the periplasmic signaling sequence, was cloned, and expressed intracellularly with highest enzymatic activity achieved by a MUT^+^ clone cultured in BMM expression medium; a value 5-fold greater than that obtained by native L-asparaginase in *P. sizovae* cells. To the best of our knowledge, this is the first literature report of the heterologous production of an L-asparaginase from a filamentous fungus by a yeast.

## 1. Introduction

Recombinant DNA technology and protein engineering provide the production of enzymes tailored to meet the needs of users or the process, obtaining enzymes of superior quality to natural properties. Cheaper, easier, and faster protein expression can be performed in prokaryotic systems such as *Escherichia coli*, however, eukaryotic proteins can be toxic to bacteria and this bacterium cannot express high-molecular-weight proteins, in addition to not being the system of choice for proteins rich in S-S and proteins that require post-translational modifications, as they cannot carry out glycosylation and remove the S–S sequences [1,2]. Yeasts, unicellular eukaryotic fungal organisms, are often used to produce recombinant proteins that do not produce well in *E. coli* due to folding problems or the need for glycosylation. The advantages of yeast expression systems include: high throughput; stable production strains; durability; cost benefit; high density growth; high productivity; suitability for isotopically labeled protein production; rapid growth in chemically defined media being easily adapted to fermentation processes; mammalian cell-like product processing; proteins with molecular weight higher than 50 kDa can be produced; signal sequences can be removed; can handle S–S-rich proteins promoting disulfide bond formation; produce chaperones to aid in the folding of certain proteins; and can glycosylate proteins [1,3]. The two most used yeasts are *Saccharomyces cerevisiae* and the methylotrophic yeast *Pichia pastoris*, renamed as *Komagataella pastoris* or *K. phaffii* [1]. Methylotrophic yeasts can use methanol as the only source of carbon and energy. The enzyme alcohol oxidase (AOX; EC 1.1.3.13) catalyzes the first step in the methanol utilization pathway: the oxidation of methanol to formaldehyde and hydrogen peroxide. The AOX is sequestered within the peroxisome along with catalase, which degrades hydrogen peroxide into oxygen and water. Part of the formaldehyde generated leaves the peroxisome and is oxidized to formate by formaldehyde dehydrogenase and carbon dioxide by formate dehydrogenase, of which these reactions are a source of energy for cells growing in methanol [4].

L-asparaginase (ASNase; EC 3.5.1.1), listed as a cytotoxic medicine for acute lymphoblastic leukemia (ALL) in the WHO Model List of Essential Medicines for children and adults [5,6], is used as a standard remission induction chemotherapy treatment option for newly diagnosed ALL cases and as a direct systemic chemotherapy prophylaxis of the central nervous system for standard-risk and high-risk ALL patients [7]. L-asparaginase catalyzes the hydrolysis of L-asparagine into L-aspartic acid and ammonia. This enzyme is widely distributed among plants, animals and microorganisms, however microorganisms are efficient and inexpensive sources of L-asparaginase as they can be cultured easily using cheap substrate and enzyme extraction and purification is convenient thus facilitating large-scale production [8]. The earliest reports of L-asparaginase discovered in bacteria showed that *E. coli* cells possess one or two asparaginase activities due to two distinct enzymes which differ in their affinities for L-asparagine, where the enzyme with the higher affinity (asparaginase II, located in the periplasmic region) markedly inhibited lymphomas in mice, while the asparaginase with the lower affinity (asparaginase I, found in the cytoplasm) was ineffective [9,10]. Only asparaginase II is used for clinical application [11], and industrialized enzyme preparations are obtained from bacteria, such as the enzyme derived from *E. coli* in native and pegylated forms and the enzyme derived from *Dickeya chrysanthemi* (syn. *Erwinia chrysanthemi*) [12]. However, adverse effects such as hypersensitivity, coagulation disorders, pancreatitis, hyperglycaemia, hepatotoxicity and resistance to L-asparaginase with antibody formation in children with leukemia and lymphoma as well as in adults undergoing induction treatment for ALL when *E. coli* and *D. chrysanthemi* asparaginase were administered have been reported [13]. Another factor that contributes to asparaginase-associated toxic side effects is its L-glutaminase activity, that is, in addition to catalyzing the hydrolysis of L-asparagine, the enzyme also catalyzes the hydrolysis of L-glutamine to L-glutamate and ammonia, which has been implicated in immunosuppression, hepatotoxicity, pancreatitis, and coagulation dysfunction [14]. Therefore, from a quality perspective, a reduction on glutaminase activity is desirable [11]. As the main reason for the interruption of L-asparaginase treatment is the development of hypersensitivity, caused partly by the protein’s high molecular weight and bacterial origin, an enzyme that leads to fewer side effects could be found in other microbial sources such as fungi, which are eukaryotic microorganisms evolutionarily closer to human beings in comparison to bacteria [15,16,17]. Furthermore, this biopharmaceutical of great pharmaceutical and industrial importance has an increasing demand in the international market that cannot be fulfilled by natural resources, thus bulk quantities of enzymes and proteins can be produced by recombinant DNA technology [18]. The heterologous expression of L-asparaginase from some microorganisms has already been reported in the literature [18,19,20,21,22,23,24,25,26,27,28,29,30,31,32,33,34,35,36,37,38]. Among the studies of fungal L-asparaginase produced recombinantly, such as *Aspergillus oryzae* [32], *A. niger* and *A. terreus* [36], which have been used in food industry for reduction of acrylamide formation in some foods [39,40], no study has reported the cloning and expression of a fungal L-asparaginase in yeast to date. Therefore, the present work aims to identify the L-asparaginase gene sequence and produce L-asparaginase from *Penicillium sizovae*, a filamentous fungus recently reported as an L-asparaginase producer with low glutaminase activity isolated from the soil of the Brazilian Savanna (Cerrado) [41], in recombinant yeast *K. phaffii* as an alternative to bacterial asparaginases in an attempt to reduce such adverse effects for ALL treatment with greater enzyme yield to meet the market demand.

## 2. Results

### 2.1. Identification of L-Asparaginase Gene Sequence from P. sizovae

The L-asparaginase gene sequences of *P. citrinum* DSM1997, *P. citrinum* JCM22607 and *P. steckii* MLKD01000003 were identified from the complete genomes by homology to L-asparaginase gene sequences already described in other species and published in the database. Thus, degenerate primers were designed and tested in the amplification of regions of the genomic DNA of *P. sizovae*, due to the taxonomic proximity of this species to *P. citrinum* and *P. steckii*. The PCR performed with degenerate primers designed from the identified L-asparaginase sequences of *P. citrinum* and *P. steckii* revealed the amplification of different regions of the *P. sizovae* genome, including L-asparaginase gene sequence, from which PCR products were obtained with the expected sizes, purified, and sequenced. The *P. citrinum* and *P. steckii* gene sequences were aligned with sequenced *P. sizovae* amplicons (Figure S1) and the L-asparaginase gene sequence from *P. sizovae* was identified (Figure S2).

### 2.2. Prediction of the Molecular Structure and Active Site Insights of L-Asparaginase from P. sizovae

The predicted nucleotide sequences of the native and partial *P. sizovae* L-asparaginase were translated, with the removal of introns, into amino acid sequences that constitute this protein (Figure S3). The computation of *P. sizovae* L-asparaginase theoretical pI is 4.93 and its theoretical molecular mass is 40073.46 Da. As a tetrameric protein, it is estimated that its molecular mass is about 160.292 kDa.

The protein sequences of L-asparaginases were analyzed and revealed that the filamentous fungus *P. sizovae* contains an enzyme with homology to its bacterial representatives. When performing an amino acid sequence alignment, protein-protein, in the database of non-redundant proteins (nr), the highest identity (95.29%) found was with a hypothetical *P. steckii* protein. This identity is related to the number of amino acids in common between the two sequences, and in the case of L-asparaginase from *P. steckii*, this had not yet been identified until the present study. All other major proteins found in the non-redundant protein database are in the same situation. Even the phylogenetic tree of this specific sequenced protein does not demonstrate proximity to other proteins that have already been identified beyond the prediction based on genomic sequences in this database.

**Figure 1:**
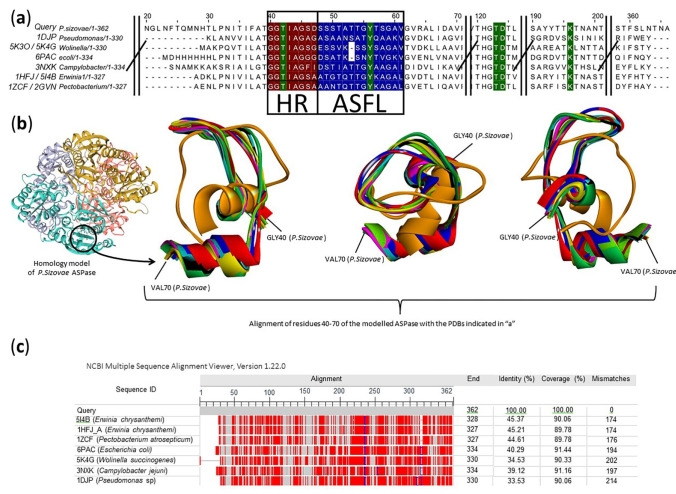
Hinge Region (HR), Active Site Flexible Loop (ASFL) and active site residues alignment. (**a**) Alignment of the most similar Protein Data Banks’s (PDBs) sequences. HR is indicated in brown, ASFL in blue and the residues relevant to the catalytic activity are highlighted in green. (**b**) Different views of the flexible region of the L-asparaginases (ASPases) aligned as in (**a**). All sequences include the correspondent alignment to the region GLY40:VAL70 of the *P. sizovae* ASPase model. In orange, the most different structure, the PDB 5K4G “open/inactive” form of *Wolinella succinogenes* ASPase. Chains: *P. sizovae* (black); *Pseudomonas* sp. (light green, PDB 1DJP); *Wolinella succinogenes* “closed/active” form (red, PDB 5K3O); *Campylobacter jejuni* (brown, PDB 3NXK); *Erwinia crysanthemi* (pink and light blue, PDBs 1HFJ and 5I4B); *Pectobacterium* (*Erwinia*) *carotovora* (yellow and green, PDBs 1ZCF and 2GVN). (**c**) Identity percentage and full alignment residue differences (in red) among the compared asparaginases.

The comparison with proteins that have already been better studied, with some structural characteristics already elucidated, was carried out for purposes of determining secondary and tertiary structure. A decrease in sequence similarity was observed, however, among the already crystallized proteins, the closest are those with L-asparaginase or glutaminase activity, which denotes conservation of the catalytic site. Besides the binding site, two relevant regions to the enzymatic activity (Hinge Region—HR and the Active Site Flexible Loop—ASFL) have been compared among other L-asparaginase crystallized structures (Figure 1a,b). The HR and ASFL regions are not essential to the substrate binding, but after the interaction, they present conformational changes that are responsible for the catalytic activity (conformations *cat*+ and *cat*−) [42]. The crystal Protein Data Bank (PDB) showing the most diverse tridimensional HR/ASFL regions (5K4G) was obtained from *Wolinella succinogenes* L-asparaginase in a position that the authors called “open/inactive”. The same study found the “closed/active” conformation (5K3O) structurally related to all other L-asparaginases [43]. The HR region has an asparaginase octapeptide pattern, highly conserved, and glycine-rich. The sequence GGTxyGGG is often observed (x = Ile or Leu; y = Ala or Gly) [44]. In *P. sizovae*, the HR region presented the sequence G^40^GTIAGSD^47^. The most similar already crystallized L-asparaginases showed related sequences in HR. The ASFL in *P. sizovae* asparaginase presents the sequence S^48^SSTATTGYTSGAV^61^ (Figure 1a). However, a precise frontier between the two regions is hard to define.

On the other hand, the active site of L-asparaginases is quite rigid and well-conserved. There are five catalytic residues of interest that are very well positioned. There is a threonine in the HR, a tyrosine in the ASFL and three other residues (a pair Thr-Asp and Lys) located about 64–67 and 137–140 residues apart, respectively, from the ASFL Tyr [42]. In the *P. sizovae* L-asparaginase, these residues are Thr42 (HR), Tyr56 (ASFL), Thr123, Asp124 and Lys196 (Figure 1a).

Assertions about active sites and polymeric interfaces are based on the comparison of conserved residues and compared with the known structures of already crystallized proteins. The most similar protein (PDB 1HFJ_A), which is based on a single chain, is L-asparaginase from *D. chrysanthemi* (the synonymy *Pectobacterium chrysanthemi*), a tetramer. The Swiss model, however, used another crystal structure of the same enzyme (Figures S4 and S5), from the same microorganism (PDB 5I4B) to create the homology model (Figure 1b).

Although the high conservation of the catalytic-relevant sites, the whole sequences show a medium/low similarity. All of the compared sequences present less than 50% of identity (Figure 1c), denoting the relevance of the active site conserved regions and tridimensional structures.

**Figure 2:**
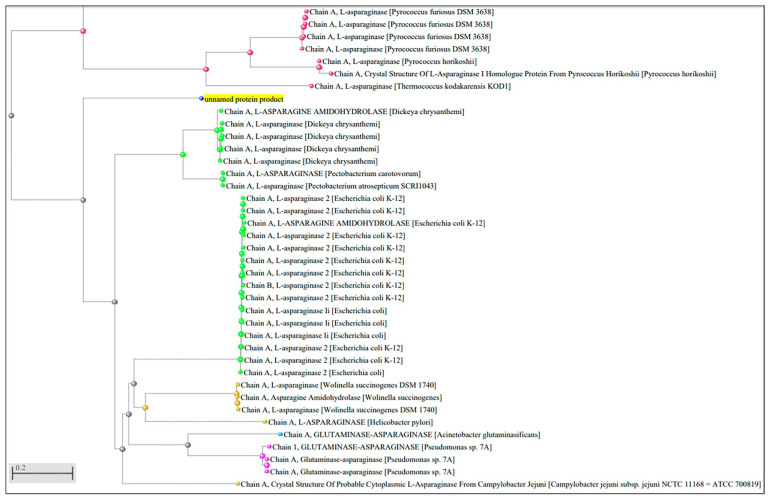
Phylogenetic tree based on the L-asparaginase sequence of *P. sizovae* compared to proteins that already have the crystallographic structure elucidated. Grishin evolutionary distance used as measure.

The crystallized L-asparaginase most similar to the sequence of *P. sizovae* is the one synthesized by the microorganism *D. chrysanthemi*. The phylogenetic tree based on the comparison of the sequenced protein of *P. sizovae* and other proteins that already have a defined crystallographic structure shows a greater evolutionary distance when compared to the proteins obtained from the non-redundant protein database (Figure 2), but it has a higher correlation with the percentage of similarity obtained with the proteins in the PDB.

### 2.3. Expression of L-Asparaginase from P. sizovae in Recombinant K. phaffii X-33

The *K. phaffii* clones were randomly selected for expression of L-asparaginase. The L-asparaginase activity was detected only in clones that integrated pPsASNase, either Mut^+^ or Mut^s^, as confirmed by direct PCR screening. The screening of culture media sought to determine the expression medium to be used in the expression of L-asparaginase where the highest enzyme activity is obtained. The highest biomass yields were produced when the selected clones were grown in BMMY culture medium, followed by MM and BMM, after 72 h of induction with methanol in the exponential phase of cell growth, a pattern observed in all tested clones.

**Figure 3:**
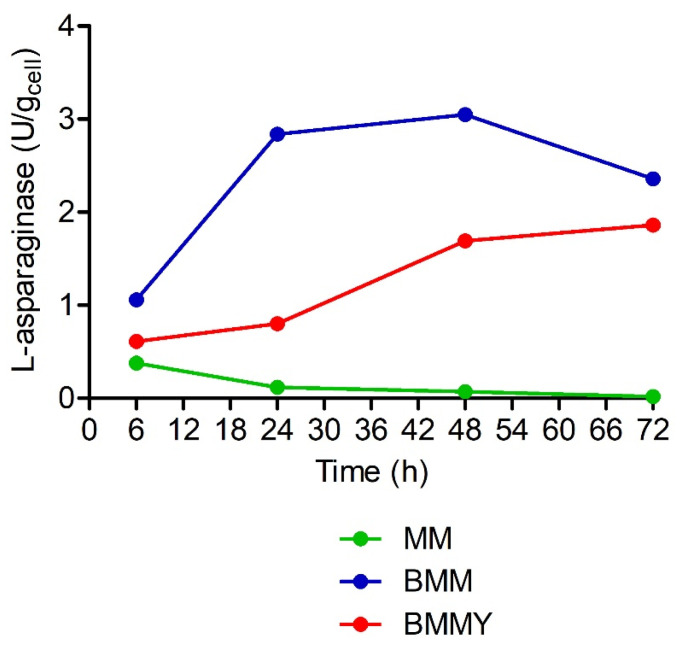
Intracellular L-asparaginase activity of a MUT^+^ clone measured in recombinant *K. phaffii* X-33 whole cell suspensions transformed with pPICZαA vector carrying partial L-asparaginase gene from *P. sizovae* cultivated in MM, BMM and BMMY expression media under methanol induction, incubated at 30 °C, 275 rpm, for 72 h.

For intracellular expression, L-asparaginase activity was detected in whole cell suspensions (intracellular activity). The highest value of intracellular L-asparaginase activity (3.05 U/g_cell_) was obtained by a clone phenotypically identified as Mut^+^ cultivated in BMM expression medium after 48 h (Figure 3).

On the other hand, for secreted expression, L-asparaginase activity was not detected in culture supernatants (extracellular activity) as expected using a pPICZαA vector. Low values of total proteins concentrations were obtained, with the highest value achieved by the selected MUT^+^ clone cultured in MM expression medium after 48 h (0.2 mg/mL), which may be a variable responsible for the lack of L-asparaginase activity quantified in the extracellular media. However, the presence of yeast extract and peptone in BMMY expression medium interfered with the total proteins assay thus overestimating the protein content for these samples. Therefore, a Sodium Dodecyl Sulfate-PolyAcrylamide Gel Electrophoresis (SDS-PAGE) of culture supernatants taken at each time point of induction (6, 24, 48 and 72 h) was performed to evaluate the presence of the secreted proteins in the extracellular medium and compared with a clone that did not integrate pPsASNase used as control. A protein band between 50 and 37 kDa was observed in all samples of the selected Mut^+^ clone (data not shown) that was not expressed by the control. As the theoretical molecular mass of *P. sizovae* L-asparaginase monomer is estimated to be approximately 40 kDa, it is suggested that this enzyme was present in the extracellular medium despite its enzymatic activity not being detected in the quantitative assay.

### 2.4. Evaluation of the Interference of Expression Media in the Quantification of L-Asparaginase Activity

**Figure 4:**
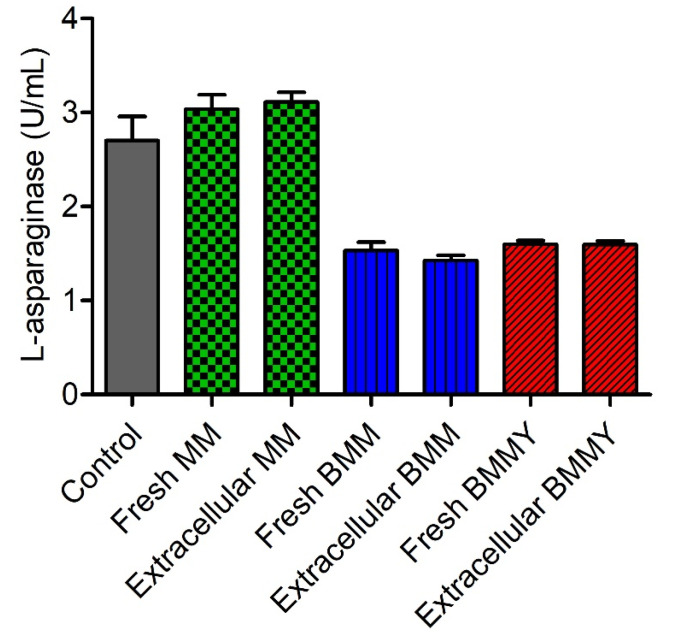
Evaluation of the interference of MM, BMM and BMMY expression media in the quantification of conditioned L-asparaginase activity using the L-aspartyl-β-hydroxamate acid method.

An enzymatic assay was carried out to verify the interference of the culture media used in the cultivation of the *K. phaffii* X-33 clones in the quantification of extracellular L-asparaginase activity using the L-aspartyl-β-hydroxamate acid method (Figure 4). There was no statistically significant difference (*p* < 0.05) between the values of L-asparaginase activity quantified in MM, BMM and BMMY expression media before and after cultivation with *K. phaffii* X-33. There was no significant difference (*p* < 0.05) between the values of L-asparaginase activity quantified in the positive control of the native enzyme and the MM expression medium. However, there was a significant reduction in the quantification of enzyme activity between the positive control and the BMM and BMMY expression media. The values were reduced to approximately half of those quantified in the control sample, which demonstrated that the expression media underestimated the enzymatic activity when the L-aspartyl-β-hydroxamate acid method was employed to quantify L-asparaginase in the extracellular medium for secreted expression.

## 3. Discussion

The L-asparaginase gene sequences of *P. citrinum* and *P. steckii* were identified from their whole genome sequences. The phylogenetic proximity of these *Penicillium* species that belong to section *Citrina* led to the identification of L-asparaginase from *P. sizovae*, a filamentous fungus isolated from the soil of the global biodiversity hotspot Cerrado in Brazil. This sequence showed homology to another L-asparaginase gene from a novel *P. cerradense*, a filamentous fungus isolated from the Brazilian Savanna, recently identified by this research group [45].

Comparison with crystallized proteins that have been deposited in databases was carried out to determine the secondary or tertiary structure of L-asparaginase from *P. sizovae*, where the closest ones are those with L-asparaginase or glutaminase activity, which denotes conservation of the catalytic site and the other regions relevant to the catalytic activity. The crystallized L-asparaginase most similar to the *P. sizovae* sequence is the one synthesized by the microorganism *D. chrysanthemi*. From the observed degree of conservation of active sites in these structures, it can be inferred that L-asparaginase from *P. sizovae* has type II asparaginase activity (periplasmic, with greater L-asparaginase activity in relation to glutaminase activity) and that it is a dimer-dimer, that is to say a tetramer, with a theoretical molecular mass estimated as 160 kDa, similar to those purified from *Citrobacter* 166 kDa [46], *E. carotovora* 160 kDa [47] and *F. tricinctum* 161/170 kDa [48].

The pPICZαA vector with the α factor for secreted expression of proteins was selected for insertion of L-asparaginase gene from *P. sizovae* in its partial sequence, lacking the first 20 amino acids, transformed into *K. phaffii* X-33. However, L-asparaginase was expressed intracellularly by the screened clones, with the highest level of enzyme activity achieved in a selected Mut^+^ clone cultured in BMM expression medium, a value 5-fold higher than native L-asparaginase quantified in cells of *P. sizovae* grown in a modified Czapek Dox medium at 30 °C and 120 rpm for 120 h (0.60 U/g_cell_) [41]. L-asparaginase was not detected by the enzymatic assay in the extracellular medium despite the removal of the first 20 amino acids that direct the enzyme to the periplasmic space and the addition of the *S. cerevisiae* α-factor signal, which is commonly used to direct the secretion of heterologous proteins in *P. pastoris*. Similarly, Ferrara et al. cloned and expressed *S. cerevisiae* periplasmic asparaginase II, which is coded by the ASP3 gene, in the *P. pastoris* expression system under the control of the promoter P_AOX1_. In their study, asparaginase activity was measured in whole cell suspensions (periplasmic activity) and in culture supernatants (extracellular activity) based on asparagine hydrolysis (conversion of asparagine into aspartate and ammonia) and asparagine hydroxylaminolysis (conversion of asparagine and hydroxylamine into β-aspartohydroxamate and ammonia) catalyzed by asparaginase. Enzyme yield per dry cell mass for the recombinant *Pichia* strain was 7-fold higher than that obtained for *S. cerevisiae*. Despite the secretion signal sequence, the assays using whole cell suspensions showed that the enzyme was addressed to the periplasmic space, no asparaginase activity was detected in the culture supernatant [27]. The use of molecular biology tools for secretion can also result in protein accumulation in the periplasm of the cell, therefore, further procedures are necessary for the protein release from the cell mass after the cell cultivation step through microbial cell disruption, aiming for high extraction yields and high enzyme recovery [49]. In the present study, *K. phaffii* cells were not subjected to any method of cell disruption, therefore the extraction of asparaginase from the yeast periplasmic space should be studied. Comparably, Rodrigues et al. cloned and expressed the same ASP3 gene in *P. pastoris* and observed only the periplasmic L-asparaginase activity, measured in whole cell suspension, based on asparagine hydroxylaminolysis. The extracellular enzyme activity was null despite its presence confirmed by SDS-PAGE [50], similar to that observed in the present study. Proteins generally require specific helper proteins, chaperones, to aid in their correct folding and to protect them from denaturation and aggregation. It is likely that chaperones conserved within the periplasmic space would be responsible for protecting proteins against folding stress [51]. The efficiency of secretion depends not only on the presence of motifs that direct the heterologous protein to the culture medium, but also on the nature of the protein’s structure. As L-asparaginase II is a periplasmic enzyme, it is possible that an unidentified domain of its protein structure may interact with the cell wall, preventing the secretion of the enzyme [27]. Therefore, it is possible that the enzyme secreted in the extracellular environment by *P. pastoris* may have undergone some modification to an inactive form [50]. Roldán et al. evaluated secreted expression in Glycoswitch^®^ using two constructs from different strains containing the asnB gene that codes for L-asparaginase from *E. chrysanthemi* with and without His-tag, in which three-dimensional modeling of the protein suggests that additional structures (His-tag) can adversely affect the native conformation and folding of L-asparaginase and, therefore, the expression and cellular secretion of this enzyme [52]. Additionally, the BMM and BMMY expression media interfered with the quantification of L-asparaginase activity when the L-aspartyl-β-hydroxamate acid method was employed, which could contribute for the low values of enzyme activity in the extracellular medium of the evaluated clones. Furthermore, the optimization of the culture medium should be investigated to improve protein expression and increase enzyme yield.

The data obtained in this work are relevant to pave the way in search for an understanding of how the mechanism for secreted expression of L-asparaginase from filamentous fungi in *K. phaffii* expression system works. As previous reports of asparaginase genes derived from yeast cloned and expressed by other yeast have shown, the removal of the original L-asparaginase signal sequence and addition of the *S. cerevisiae* α-factor signal were not sufficient to secrete the enzyme from a filamentous fungus by a yeast in measurable amounts and in catalytically active form. However, our data show that partial L-asparaginase from *P. sizovae* was successfully cloned and expressed intracellularly, and future work should focus on the purification and determination of enzymatic activity, comparing it to the activities of the commercially available bacterial enzymes and evaluate their biological properties.

## 4. Materials and Methods

### 4.1. Microorganisms and Plasmids

A fungal isolate from the Brazilian Savanna soil that has been previously identified as an L-asparaginase producer with low glutaminase activity [41], *P. sizovae*, was used as a source of genomic DNA. *Escherichia coli* One Shot™ TOP10 was used for plasmid amplification and other DNA manipulations. Wild-type *P. pastoris* X-33 (Thermo Fisher Scientific, Waltham, MA, USA), also known as *K. phaffii* [53], was used as a host for L-asparaginase gene expression. pPICZαA was used for cloning the PCR product and pPIC9 (Thermo Fisher Scientific) was used as the expression plasmid in *K. phaffii*.

### 4.2. Culture Media

Low Salt Luria-Bertani Medium (LB): 1% Tryptone water; 0.5% Yeast Extract; pH 7.5; Zeocin™ to 25 μg/mL final concentration.

Low Salt LB Agar Plates: identical to Low Salt LB medium with 15 g/L agar.

Yeast Extract Peptone Dextrose Medium (YPD): 1% yeast extract; 2% peptone; 2% dextrose (glucose).

YPD + Zeocin™ Medium: identical to YPD with or without 2% agar; 100 μg/mL Zeocin™.

YPDS + Zeocin™ Agar: identical to YPD + Zeocin™ with 1 M sorbitol.

Minimal Glycerol (MGY) and Minimal Methanol (MM): 1.34% Yeast Nitrogen Base (YNB); 4 × 10^–5^% biotin; 1% glycerol or 0.5% methanol.

Buffered Minimal Glycerol (BMG) and Buffered Minimal Methanol (BMM): 100 mM potassium phosphate, pH 6.0; 1.34% YNB; 4 × 10^–5^% biotin; 1% glycerol or 0.5% methanol.

Buffered Glycerol-complex Medium (BMGY) and Buffered Methanol-complex Medium (BMMY): 1% yeast extract; 2% peptone; 100 mM potassium phosphate, pH 6.0; 1.34% YNB; 4 × 10^−5^% biotin; 1% glycerol or 0.5% methanol.

### 4.3. Identification of L-Asparaginase Gene Sequence from P. sizovae

*Penicillium sizovae* was grown in a Petri dish containing PDA culture medium kept at 32 °C for 6 days. The mycelium was carefully scraped with a disposable scalpel, transferred to a 2 mL centrifuge tube with zirconia beads (200 µL) and vigorously shaken in FastPrep FP120 (Savant Instruments, Hyderabad, India) at 6.0 speed, for 4 cycles of 30 s pulse with intervals between pulses to avoid overheating and consequent sample degradation.

After extraction, *P. sizovae* genomic DNA was purified using the Plant/Fungi Isolation Kit (Norgen BioTek Corporation, Thorold, Canada), diluted 10 times with nuclease-free water and quantified in triplicate with an aliquot of 1 µL in a DS-1 FX Spectrophotometer (DeNovix, Wilmington, DE, USA) to assess the concentration and purity of the DNA, which was verified by the absorbance ratios at 260/280 nm and 260/230 nm. Two primers were used in a polymerase chain reaction (PCR) that amplify the Internal Transcribed Spacer (ITS) regions ITS1 (5′-TCCGTAGGTGAACCTGC-3′) and ITS4 (5′-TCCTCCGCTTATTGATATGC-3′) [54,55]. The PCR was performed with 3 µL template DNA, 1 µL of each primer (0.2 µM), 1 µL of dNTPs (200 µM), 0.25 µL of *Taq* polymerase (1.25 U), 5 µL of 1X *Taq* standard buffer (New England Labs) and 38.75 µL nuclease-free water in a total 50 µL reaction volume. The thermal program included an initial denaturation at 95 °C for 5 min, followed by 30 cycles at 95 °C for 30 s, annealing at 55 °C for 30 s, initial and final extensions at 68 °C for 45 s and 5 min, respectively. The purity of the samples was verified on a 1.2% agarose gel and gDNA samples were stored at −20 °C.

A search in the GenBank (https://www.ncbi.nlm.nih.gov/genbank/ (accessed on 27 January 2019)) database for L-asparaginase gene sequences from fungal species of the genus *Penicillium* was performed, which retrieved the complete genome of three fungal strains—*Penicillium citrinum* DSM1997, *Penicillium citrinum* JCM22607 and *Penicillium steckii* MLKD01000003-phylogenetically close to *P. sizovae* species (all species of the section *Citrina* according to the phylogeny of the genus *Penicillium*) [56]. The complete genome of these strains was compared using the ClustalX 2.1 software with L-asparaginase type II gene sequences already identified in other fungal species and deposited in the GenBank database, thus determining the L-asparaginase gene sequences of these fungi. The alignment of the identified L-asparaginase sequences revealed the presence of non-homologous nitrogenous bases between the fungal species *P. citrinum* and *P. steckii*. For this reason, degenerate primers comprising different stretches of the L-asparaginase gene-sequence prior to the start of the L-asparaginase gene, flanking regions, introns, and sequence that follows the L-asparaginase gene-were designed and synthesized (Eurofins Genomics UK Limited), out of which 4 forward (F1.2, F2, FASP2 and FASP3) and 3 reverse (RASP2.1, RASP6 and R2) primers were used to identify *P. sizovae* L-asparaginase gene (Table 1).

**Figure 5:**
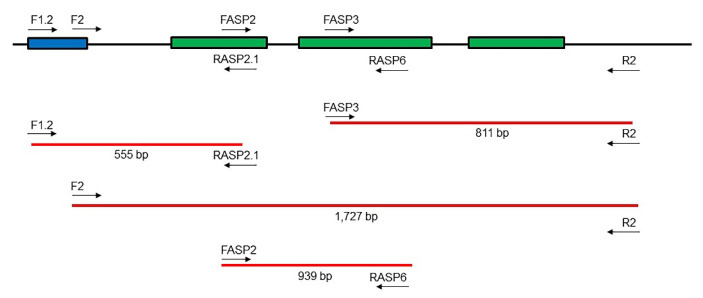
Synthesized degenerate primers comprising sequence prior to the start of the L-asparaginase gene (blue), flanking regions, L-asparaginase gene (green) with introns, sequence posterior to the L-asparaginase gene and the alignment of primers for the identification of the L-asparaginase gene from *P. sizovae* (red).

Pairs of forward and reverse primers were tested in PCR for the amplification of different regions where the L-asparaginase gene is found in *P. sizovae* genome: F1.2/RASP2.1 (555 bp), F2/R2 (1727 bp), FASP3/R2 (811 bp) and FASP2/RASP6 (939 bp) (Figure 5). The PCR was performed with 3 µL template DNA, 2 µL of each primer (0.5 µM), 25 µL of 2X Phusion Green Hot Start II High Fidelity Master Mix (Thermo Fisher Scientific) and 18 µL nuclease-free water in a total 50 µL reaction volume. The thermal program included an initial denaturation at 98 °C for 30 s, followed by 30 cycles at 98 °C for 10 s, annealing at 52 °C for 30 s, initial and final extensions at 72 °C for 1:10 min and 10 min, respectively. The purity of the samples was verified on a 1.2% agarose gel.

After confirming the amplification, the PCR products were purified using the GeneJet PCR Purification Kit (Thermo Fisher Scientific) and visualized on a 1.2% agarose gel using 5 µL of each sample and 2 µL of gel loading buffer. For sequencing the amplified regions of the *P. sizovae* genome that comprise the L-asparaginase gene, 2 µL of each primer (forward or reverse) were added to 15 µL of their respective purified PCR products and sent for sequencing by Eurofins Genomics using the Mix2Seq kit. The sequence of each stretch of the amplified and purified PCR products was aligned by homology to the L-asparaginase sequences identified according to the complete genome of *P. citrinum* and *P. steckii* using software ClustalX 2.1. The identified L-asparaginase gene sequence (reverse complement) was deposited in the GenBank database under accession number MW291568.

### 4.4. Prediction of the Molecular Structure and Active Site Insights of L-Asparaginase from P. sizovae

The nucleotide sequences of the two isoforms of L-asparaginase from *P. sizovae*—native and partial—were translated into their respective amino acid sequences through ExPASy Bioinformatics Resource Portal. The isoelectric point and the molecular mass were computed through the insertion of the respective protein sequences in the “Compute pI/Mw tool” of Swiss-Model ExPASy platform. Additionally, the three-dimensional molecular structures were designed using *D. chrysanthemi* 5I4B PDB as the homology template (the best-matched PDB structure found by the Swiss-Model application). An alignment of the amino acid sequence, protein-protein, in the non-redundant (nr) protein database was performed. The Hinge Region (HR) and Active Site Flexible Loop (ASFL), essential regions to the stabilization of the catalytic site [42,44], were aligned and compared.

### 4.5. Preparation of Recombinant Plasmid

**Figure 6:**
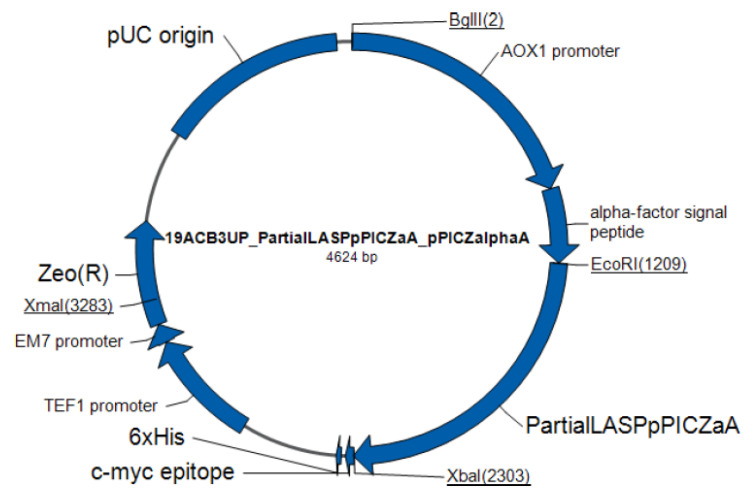
Plasmid map of pPICZαA carrying the synthetic partial L-asparaginase gene from *P. sizovae*.

EasySelect™ *Pichia* Expression Kit (Invitrogen™ by Life Technologies™) was used for expression of L-asparaginase from *P. sizovae* using pPICZα in recombinant *K. phaffii* X-33. The synthetic partial L-asparaginase gene from *P. sizovae* (pPsASNase) was assembled from synthetic oligonucleotides and/or PCR products by Invitrogen (Thermo Fisher Scientific). The plasmid (Figure 6) was built with the promoter P_AOX1_ that allows methanol-inducible, high-level expression in *Pichia*, a native *S. cerevisiae* α-factor secretion signal that allows for efficient secretion of most proteins from *Pichia*, the Zeocin™ resistance gene, and the restriction sites EcoRI and XbaI at the 5’ end and the 3’ end, respectively. The fragment, lacking the L-asparaginase first 20 codons (initiation codon and 19 codons referring to periplasmic signaling sequence), the termination codon and introns, was inserted into pPICZαA_A241 (1086 bases). The ’TA’ bases were inserted before the Xbal restriction site to frame the protein sequence with the histidine tag, which allows purification of the recombinant fusion protein on metalchelating resin. The plasmid DNA was purified from transformed bacteria, the concentration determined by UV spectroscopy and the final construct was verified by sequencing.

Transformation of competent *E. coli* cells with 1 ng of pPsASNase was performed by a chemical method using One Shot™ TOP10 Chemically Competent *E. coli* (Invitrogen Thermo Fisher Scientific) according to the manufacturer’s instructions. Transformation of 10 pg of pUC19 was used as control. The transformation mix was plated onto Low Salt LB agar plates supplemented with 25 µg/mL Zeocin™. For the pUC19 control, the transformation mix was diluted 1:10 in LB culture medium and added to LB agar plates supplemented with 100 µg/mL ampicillin. Plates were incubated at 37 °C overnight. Zeocin™-resistant transformants were picked and inoculated into Low Salt LB liquid medium with 25 µg/mL Zeocin™ and grown overnight at 37 °C with shaking. Plasmid DNA was extracted from transformed *E.* coli cells using QIAGEN^®^ Plasmid Maxi Kit (Qiagen Strasse) according to the manufacturer’s instructions and the construct was sequenced to confirm that the gene is in frame with the C-terminal peptide. The pDNA (5 µg) was linearized with Anza™ SAC I restriction enzyme (Invitrogen Thermo Fisher Scientific) followed by heat inactivation according to the manufacturer’s instructions. Ethanol precipitation of pDNA was carried out using 1/10 volume 3 M sodium acetate and 2.5 volumes of 100% ethanol. The mixture was incubated at −80 °C for one hour then centrifuged at 12,000× *g* for 15 min at 4 °C. The pDNA pellet was washed with 70% ethanol, centrifuged, air-dried, and resuspended in 5 µL nuclease-free water (1 µg/µL).

### 4.6. K. phaffii X-33 Transformation and Phenotypic Screening

Transformation of competent *K. phaffii* X-33 cells with 5 µg of linearized and precipitated pPsASNase was performed by a chemical method using *Pichia* EasyComp™ Kit (Invitrogen™ Life Technologies) according to the manufacturer’s instructions. The transformation mix was plated onto YPDS with 100 μg/mL Zeocin™ agar plates and incubated for 3 to 10 days at 30 °C. Zeocin™-resistant transformants were selected, transferred to new Petri dishes containing YPD agar to isolate Zeocin™ resistant transformants and the Mut phenotype was confirmed by direct PCR screening of *K. phaffii* clones. A patch of selected colonies was resuspended in 10 µL of 0.02 M NaOH following incubation at 95 °C for 10 min. One microliter of the gDNA was used in a PCR as described previously with 2X Phusion Green Hot Start II High Fidelity (Thermo Fisher Scientific) in a total 20 µL reaction volume using 5′AOX and 3′AOX primers (Invitrogen™). In order to confirm the extraction of gDNA from the clones, ITS1 and ITS4 primers were used in PCR as a positive control. Samples were visualized on a 0.8% agarose gel.

### 4.7. Screening of Transformants and Expression of L-Asparaginase

The *K. phaffii* X-33 clones containing pPsASNase were selected for enzyme expression in liquid medium. An initial screening was conducted to evaluate three culture media used for cell growth (MGY, BMGH, BMGY) followed by three culture media used for induction (MM, BMM and BMMY, respectively) of L-asparaginase expression. A single colony of the selected clones was inoculated into 10 mL of MGY, BMG, and BMG in a 50 mL centrifuge tube at 30 °C and 275 rpm in a shaking incubator for approximately 40 h. Cells were harvested by centrifuging at 1500–3000× *g* for 5 min at room temperature. The supernatant was discarded, and the cell pellet was resuspended in 25 mL of MM, BMM and BMMY culture media, respectively, in previously autoclaved 250 mL flasks to induce expression. Methanol (100%) was added to a final concentration of 0.5% every 24 h to maintain induction. Aliquots of the expression culture (1 mL) were transferred to 1.5 mL microcentrifuge tubes after 6, 24, 48 and 72 h. Samples were centrifuged at 13,000× *g* for 2–3 min at room temperature. The supernatant was transferred to new 1.5 mL tubes to assess the secreted expression of the enzyme in the culture medium, while the cell pellet was used to assess the intracellular expression of the enzyme. Tubes containing *K. phaffii* cell pellets were weighed to quantify the biomass produced at different time points. The culture supernatants and the cell pellets were stored at −80 °C until ready to assay. L-asparaginase activity was quantified in the cells and in the culture supernatants to assess intracellular and secreted protein expression, respectively, while the quantification of total proteins and visualization of proteins by SDS-PAGE was performed only in the culture supernatants to assess secreted protein expression. Total proteins quantification was performed using the Pierce™ BCA Protein Assay Kit (Thermo Fisher Scientific) according to the manufacturer’s instructions. SDS-PAGE was performed as described by Laemmli [57] in 12% polyacrylamide gel and stained according to Blum et al. [58] using the PlusOne Silver Staining Kit (GE Healthcare), an ultra-sensitive method for detection of proteins. Culture supernatants of BMMY expression medium taken at each time point of induction (6, 24, 48 and 72 h) of a selected *K. phaffii* X-33 clone that integrated pPsASNase, in which the highest intracellular L-asparaginase activity was obtained, were analyzed and compared with a *K. phaffii* X-33 clone that did not integrate pPsASNase, confirmed by PCR screening and null enzyme activity, cultured under the same conditions in BMMY expression medium after 72 h of induction and used as control.

### 4.8. L-Asparaginase Activity Assay

L-asparaginase activity can be determined by quantifying the β-hydroxamate aspartic acid produced by the hydroxylaminolysis reaction carried out in the presence of hydroxylamine. The quantification of L-asparaginase was measured in whole cell suspensions (intracellular activity) for determination of intracellular expression and in culture supernatants (extracellular activity) for determination of secreted expression by the L-aspartyl-β-hydroxamic acid (AHA) method described by Drainas et al. [59] with modifications. The reaction mixture for intracellular protein expression consisted of *K. phaffii* cells obtained in 1 mL culture, 750 µL of 50 mM Tris-HCl buffer pH 8.6, 100 µL of 100 mM stock L-asparagine solution (final concentration 10 mM) and 100 µL of 1 M stock hydroxylamine solution (final concentration 100 mM) previously neutralized with 2 M NaOH. The reaction mixture was incubated at 37 °C for 30 min. 250 µL of ferric chloride reagent (10% (*w*/*v*) FeCl3 and 20% (*w*/*v*) trichloroacetic acid in 0.66 M HCl) was added (final volume 1.25 mL). The reaction mixture for secreted protein expression consisted of 80 µL of culture supernatant, 80 µL of 50 mM Tris-HCl buffer pH 8.6, 20 µL of 100 mM stock L-asparagine solution (final concentration 10 mM) and 20 µL of 1 M stock hydroxylamine solution (final concentration 100 mM) previously neutralized with 2 M NaOH. The reaction mixture was incubated at 37 °C for 30 min. Then 50 µL of ferric chloride reagent was added (final volume 250 µL). The samples were centrifuged at 5000 rpm for 5 min at 4 °C. The supernatant (200 µL) was transferred to a 96-well microplate and the absorbance was measured at 500 nm in a microplate reader (Synergy HT, BioTek Instruments, Winooski, VT, USA). An L-aspartic acid β-hydroxamate standard curve (0–0.3 µmol) was constructed with 50 mM Tris-HCl buffer pH 8.6 and 50 µL ferric chloride reagent, in triplicate. The obtained equation was y = 2.6242x + 0.0025, R^2^ = 0.9999, where y is the sample absorbance and x is the L-aspartic acid β-hydroxamate formed in µmol. One unit of L-asparaginase was defined as the amount of enzyme needed to form 1 µmol of L-aspartic acid β-hydroxamate per minute per gram of cell or mL of culture medium as shown in Equation (1).

(1) $$\mathrm{Enzyme}\;\mathrm{activity}\;=\frac{\left(µ\mathrm{mol}\;\mathrm{of}\;L\;-\;\mathrm{aspartic}\;\mathrm{acid}\;\beta \;-\;\mathrm{hydroxamate}\;\mathrm{formed}\right)}{\left(\min\mathrm{ofincubation}\;\times \;g\;\mathrm{of}\;\mathrm{cells}\;\mathrm{taken}\;\mathrm{or}\;\mathrm{mL}\;\mathrm{of}\;\mathrm{culture}\;\mathrm{supernatant}\right)}$$

### 4.9. Evaluation of the Interference of Expression Media in the Quantification of L-Asparaginase Activity

An enzymatic assay was carried out to verify the interference of the expression media used in the cultivation of *K. phaffii* X-33 clones on the quantification of L-asparaginase activity using the L-aspartyl-β-hydroxamate acid method. The crude extract of the fungus *P. sizovae* was prepared as described by Freitas et al. [41]. The native enzyme in the crude extract was used in all samples and as a positive control of the experiment. The expression media used—MM, BMM and BMMY—were evaluated before and after cultivation with *K. phaffii* X-33 cells by adding the same amount of enzyme to all samples, in triplicate. The reaction mixture consisted of 70 µL of 50 mM Tris-HCl buffer pH 8.6, 10 µL of *P. sizovae* crude extract, 80 µL of expression media (fresh or previously cultivated with *K. phaffii* cells), 20 µL of 100 mM stock L-asparagine solution and 20 µL of 1 M stock hydroxylamine solution. The reaction mixture was incubated at 37 °C for 30 min and 50 µL of ferric chloride reagent was added. The reaction mixture for the positive control was the same as described previously, replacing the volume of expression media with Tris-HCl buffer (150 µL total). The results are presented as mean ± standard deviation of enzyme activity. Statistical analysis was assessed by 1way ANOVA in GraphPad Prism.

## 5. Conclusions

This work identified the L-asparaginase gene sequence from *Penicillium sizovae*, a filamentous fungus isolated from the Brazilian Savanna (Cerrado) soil with low glutaminase activity, by homology to L-asparaginases from *Penicillium* species of the section *Citrina*: *P. citrinum* and *P. steckii*. To determine the secondary or tertiary structure of L-asparaginase from *P. sizovae*, comparison with crystallized proteins revealed the closest ones are those with L-asparaginase or glutaminase activity, which denotes conservation of the catalytic site and other regions relevant to the catalytic activity. Based on the degree of conservation observed in these structures, it can be inferred that *P. sizovae* has type II asparaginase activity (periplasmic, with greater L-asparaginase activity in relation to glutaminase activity) and that it is a tetramer with a theoretical molecular mass estimated as 160 kDa and theoretical isoelectric point of 4.93. Partial L-asparaginase from *P. sizovae*, lacking the periplasmic signaling sequence, was cloned, and expressed by the yeast *Komagataella phaffii* X-33 intracellularly. The highest value of enzymatic activity was achieved by a MUT^+^ clone cultured in BMM expression medium with a value 5-fold greater than that obtained by native L-asparaginase in *P. sizovae* cells, thus biosynthesizing higher yields of this enzyme. L-asparaginase was not detected in the extracellular medium as expected, despite the removal of the first 20 amino acids that direct the enzyme to the periplasmic space and the addition of the *S. cerevisiae* α-factor signal although its presence was suggested by SDS-PAGE, which could be due to interference of the expression media with the L-asparaginase activity assay and low protein concentration in the extracellular medium. This is the first time that the heterologous production of the enzyme L-asparaginase from a filamentous fungus by a yeast is reported in the literature, making it unprecedented, which can lead to a potential production of this enzyme with desired functional characteristics for an improvement in the therapeutic applications of ALL.

## Figures and Tables

**Table 1:** Designed and synthesized degenerate primers for the identification of L-asparaginase gene sequence from *P. sizovae*.

Primers	Sequence (5′–3′)
F1.2	AACAARGCGGAACCAACG
F2	GACARTCGRTACGATAAGC
FASP2	TGAGGGTTCAAGTATCCAC
FASP3	AGGAATCCCATTTCCATTGC
RASP2.1	TGCYAGTGGATACTTGAACC
RASP6	CAGGCTCAGATTCAAGCTC
R2	GAAGYAGTACGATAAGATCAC

R: A or G; Y: C or T.

## Data Availability

Data is contained within the article and Supplementary Material.

## Supplementary Materials

The following supporting information can be downloaded at https://www.mdpi.com/article/10.3390/ph15060746/s1. Figure S1: Nucleotide alignment of *P. citrinum* and *P. steckii* sequences to construct the L-asparaginase gene sequence of *P. sizovae* with the degenerate primers used in identification (reverse complement). Forward primers F1.2, F2, FASP2 and FASP3 (blue); reverse primers RASP2.1, RASP6 and R2 (pink); initiation codon (green); stop codon (red); Figure S2: *Penicillium sizovae* gene sequence. L-asparaginase (bold) and introns (underlined); Figure S3: Predicted amino acid sequence of the native and partial (bold) L-asparaginase gene from *Penicillium sizovae*; Figure S4: L-asparaginase crystal structure using Swiss model. Homology model prediction quality evaluation. QMQE: Global Model Quality Estimate (range 0–1); QMEANDisCo Global [60]: Model Quality Estimation with Distance Constraints; Figure S5: Alignment of L-asparaginases Conserved Protein Domain Family of type II L-asparaginases with the enzyme from *P. sizovae* (query). In yellow are the conserved residues of homotetrameric interfaces. HR: Hinge Region, ASFL: Active Site Flexible Loop. * Residues that form the active site.
