# Supplementary material for: L-Asparaginase from Penicillium sizovae Produced by a Recombinant Komagataella phaffii Strain
Source: Pharmaceuticals (Basel). 2022 Jun 14;15(6):746. doi: 10.3390/ph15060746 (PMC9227789; doi:10.3390/ph15060746)
Supplement: Supplementary file 1 [file pharmaceuticals-15-00746-s001.zip › pharmaceuticals-1734867-SI.pdf]

```
                                                           F1.2
Pcitrinum       --------GATCAGTCGACAAACTACCCAATCCCTTCCAACAAGGCGGAACCAACGCTTC
Psizovae        ------------------------------------------------------------
Psteckii        CTGAAAATGATCAATCAGCAAACTACCCAATTCCCTCCAACAAAGCGGAACCAACGCTGC
                        ***** **  ************* ** ******** ************** *

Pcitrinum       ACGATGGTCGACAGTCGCCTATTGCCGACCATGAAGGACATCTCCGTAAAGATCTACCTC
Psizovae        -------------------------------------------CCGTAAGGACCTGCCTA
Psteckii        GCGATGGCCGACAGTCACCTATTGCGGATCATGAGGGAAACCTGCGAAAGGATCTACCTG
                                                            **:**.** **.***
                                                    F2
Pcitrinum       AAGATGTAAAGGAGCACAATAAAGATATGGACAATCGATACGATAAGCCATACAATCATA
Psizovae        AGGATGTAAAGGAGCACAATAAAGATATGGACAATCGGTACGATAAGCCATACAATCATA
Psteckii        AGGATGTAAAGGAACACAATAAAGAGATGGACAGTCGGTACGATAAGCCATATAATCATA
                *.***********.*********** *******.***.************** *******

Pcitrinum       TTGCGGACGAAGGGAATATCGAAGGCGGCTGGGAGAAGAAATAAATTGGGATTGTCTGCA
Psizovae        TCGCGGACGAAGGGAATGTCGAAGGCGGTTGGGAGAAGAAATAGATTGGGACGATCTGCA
Psteckii        TTGCCGATGAAGGAAATGTCGAAAGCGGCTGGGAGAAGAAATGAATTTGATTGAATGTCG
                * ** ** *****.***.*****.**** *************..*** *.:   .:   *.

Pcitrinum       GGTATCCGCTATGTGTAGCAAGATTACACTGGTGCAAGGCCTTTTATGTCATTCAGCTTG
Psizovae        GATGTTCGTTATGTGTAGCGAGATTATACTGGTGCAAAG-TTTTTATGTCATTTAAGTTG
Psteckii        -------------------------------------------TTATGTCATTTAAATTG
                                                           ********** *. ***

Pcitrinum       GAGAATACT-------ACT---TCTGCAATCTCCCAGTTTCTCTCATAT-CACACTATTA
Psizovae        GATAATTCT-------ACTGGTTCTGCAATCTCCCTGTCTGTCTCAAAT-TATACTGTAC
Psteckii        GAAGATACATCATCTGACTGCTTCTGTGATCTCCAAGCTCCTCTCATACGGATGCTATTT
                ** .**:*:       ***   **** .******.:*    *****:*   *  .**.*:

Pcitrinum       TGTCTTACTGTCATCAATTTAGGCAAGAG-----AGGAAATAAATTAGAAAATAATAATG
Psizovae        TAGTACGTCTTTCTCCATTATGACATGGGGAATAGGGGAAGGAAAGATCAATAAATAATG
Psteckii        AGTACGTCTTTCTGTCATTGTATCGCAGGG---GGGAATAAAGTAAATAAATAACCAATG
                :.         *     .*** :. *. ..*      .*..:* ..:: *  .**::*. ****

Pcitrinum       TTCAGCGAGTTTATTCATTATTTACAGCTAATATCGATGTTTTTGTAACTACCTATCTGG
Psizovae        TTCTGCGAAGTTACTCTCTATTTACTGCTAGTATCAATGTTTTTGTAACTACCTACGTGG
Psteckii        TTCTGCGAAATTCTTCTCTATTTATGGCTAG---------------------CCATATGG
                ***:****. **. **: ******  ****.                     * *  ***

Pcitrinum       GTATGTAGGAGTCAAATCAAAGATCGGCCCATATGTTATTTGTTCTTCTTGCAACCTTCT
Psizovae        GTATGTAGGGGTCAAGTCAAAGAGTGGCCTATATGTCATCGG---TTTTTGCAATCTTCT
Psteckii        ----------ATCAAATTAAAGATCGATCTAAGTGTCATTAT------TCCCAATAGTCT
                          .****.* *****  *. * *:.*** **         *  ***  . ***

Pcitrinum       CTCTCAGAAGTTAAGACTAGGCATTTGTGTTTAGAGCAAATGTCGACGCAATCTCAGTGA
Psizovae        CTGCCAAACACTAG-TCTAGGCATTTGTATTAAGAGAAAAGGTTGAAGCAATCTCAGTGA
Psteckii        CTTCTAAATTTTAG-ACTAGGCATTTGTATTTAAAGCAAATGTTGAAGCAATTTCAGTGA
                **   *.*   **. :************.**:*.**.*** ** **.***** *******
                                                                 FASP2
Pcitrinum       TGTTGTTTGACTTCGCCAGCAATAGTCCCAGTAATATACGAGATTTTTGAGGGTTCAAGT
Psizovae        TGTTGCTCGACTTCGCTAGCAATAGTCCCAGTAATATACGGGATTTTTGAGGGTTCAAGT
Psteckii        TGTTGCTTGACTTTGCTAGCAATAGTCCAAGTAATATACGGGATTTCTGAGGGTTCAAGT
                ***** * ***** ** ************.***********.***** *************
                                                                 RASP2.1
Pcitrinum       ATCCACTGGCAATATGAGTGGCAGTGCTGCTGTCGACGTCTGACAATGGCACCTCACCAT
Psizovae        ATCCACTGGCAATATGAGTGGCACTGGTGCTTTCGACGTCTGACAATGGCACTTCACCAT
Psteckii        ATCCACTAGCAATATGAGTGGCACTGTTGCTTTCGACGTCTGACAAGGGAACTTCACCAT
                *******.*************** ** **** ************** **.** *******

Pcitrinum       TGACCGTGCGCATACTTTGAATAATTGGAATTCCAAGGCGATTGATAGCGTCTTCAATAG
Psizovae        TGACCGTACGCATACTTTGAATTATTGGGATTCCGAGGCGATTGATAGCGTCTTCAATGG
Psteckii        TCACAGTACGCATACTTTGGATTATCGGGATTCCGAGGCGGTTGATGACGTCCTCCATGG
                * **.**.***********.**:** **.*****.*****.*****..**** **.**.*

Pcitrinum       CATAGTTGAATGATGTTGTGACGCCTCCTGCTCCAGCTCCTGCAATCTACGACTAGTCAG
```

```
Psizovae        CATAGTTGAATGACGTTGTGACTCCTCCGGCCCCAGCCCCTGCAATCTACGACCAGTCAG
Psteckii        CATAATTGAATGATGTTGTCACACCTCCGGCCCCGGCCCCTGCAATCTACGAACAGTCAG
                ****.******** ***** ** ***** ** **.** **************. ******

Pcitrinum       TTATTGAACAAAATTC-ACAGAAAATGTGCTTATGTCAATTGTGAAAGTGTAACCTACCA
Psizovae        CTGTTAAACAACATTT-ACGGGAAATATTTTTCTGTCAATTCTGAAAGGATGACCTACCA
Psteckii        CTATTGAACAATTTTTGACAGAAATTGTACTTCTGCCTAATTTGAATGGATGACCTACCA
                 *.**.***** :**  **.*.**:*.*  **.** *:*:* ****:* .*.********

Pcitrinum       CGATGCCCTTTGCGCCACTATCGATGGCATTGTACAAGGTGTCATTGTGCATATCCTCGT
Psizovae        CGATGCCCTTTGCGCCACTTTCGATGGCATTGTACAAGGTGTCATTATGCATATCCTCAT
Psteckii        CAATGCCCTTTGCACCACTTTCAACCGCGTTGTATAAGGTGTCGTTATGCATATCCTCAT
                *.***********.*****:**.*  **.***** ********.**.***********.*

Pcitrinum       AAGAGAAGAGGATATCAACTCTCGGAATTTCCGTGACGTTGGCAATATCGAAGTTCTTCT
Psizovae        AAGAGAAGAGAATATCAACTCTCGGAATTTCCGTGACGTTGGCAATGTTGAAGTCCTTCT
Psteckii        AGGAGAAGAGAATATCGACTCTCGGGATTTCCTTGACGTTGGAAATATCAAAGTCCTTCT
                *.********.*****.*******.****** *********.***.*  .**** *****

Pcitrinum       TTCCCGTCGGCTGTACGGGAGGGTAGAAAAAGAATGGAGTATTAGAGATCATCTCTCCTA
Psizovae        TTCCCGTCGGCTGTACGGGGGGGTAGAAAAAGAATGGGGTATTAGAGATCATTTCTCCTA
Psteckii        TTCCCGTTGGCTGTACAGGAGGGTAGAAAAAGAATGGTGTATTAGAGATCATCTCTCCTA
                ******* ********.**.***************** ************** *******
                    FASP3
Pcitrinum       GGAATCCCATTTCCATTGCTTTAAACGTATCAATGGTATTGGCGTTGGTCTTGGTCGTGT
Psizovae        AGTATCCCATTTCCATTGCTTTGAACGTATCCATGGTGTTGGCGTTGGTCTTGGTCGTGT
Psteckii        GGAATCCCATTTCCATTGCTTTGAATGTATCCATGGTGTTAGCGTTGGTCTTAGTTGTGT
                .*:*******************.** *****.*****.**.***********.** ****

Pcitrinum       AGTAGGCGGATGCGATGCGATCATTCATGACGATCATGGCACCGCGGTTCTTGGCCTTCG
Psizovae        AGTAAGCGGATGCGATGCGATCATTCATGACAATCATGGCACCGCGGTTCTTAGCCTTCG
Psteckii        AGTAAGCGGATGCGATGCGATCATTCATAACAATCATGGCACCGCGATTCTTGGCCTTCG
                ****.***********************.**.**************.*****.*******

Pcitrinum       GGGATGCTGCGACTGTAACAGACTCAAGGAGATTGAAGGGCCCATCGGCTGAGATGGCGG
Psizovae        GAGATGCTGCGACGGTAACGGATTCAAGGAGATTGAAGGGTCCATCAGCTGAGATGGCGG
Psteckii        TAGATGCCGCGACAGTAACTGATTCGAGAAGATTGAAGGGTCCGTCGGCTGAGATGGCGG
                 .***** ***** ***** ** **.**.*********** **.**.*************

Pcitrinum       TCGAAGGGCGCATGGCGCCAACGATGATGACGGGTTTACCACAATTGATTGTAGCGTCGA
Psizovae        TTGAAGGACGCATGGCGCCAACAATGATGACGGGTTTACCACAATTGATCGTAGCGTCAA
Psteckii        TCGAAGGGCGCATAGCGCCAACAATGATAACCGGTTTACCACAATTGACCGTAGCATCCA
                * *****.*****.********.*****.** ****************  *****.** *

Pcitrinum       GGAAAAAGGCAGTTTCCTCCAAAGTGTCAGTGCCATGAGTGACAACTGCTCCAGCCATGG
Psizovae        GGAAAAAAGCAGTTTCCTCCAAAGTATCGGTTCCATGAGTAACAACAGCCCCAGCCATGG
Psteckii        AGAAAAAGGCAGTTTCCTCCAAAGTATCGGTGCCATGAGTGACAACAGCTCCAGCCATGG
                .******.*****************.**.** ********.*****:** **********

Pcitrinum       TTGGATCGTCGCAGACAAGCTTATTGATCTGCTTTGACAGTGAAATTAGAATGTCGGAGG
Psizovae        TTGGATCGTCGCAGACAAACTTGTTGATCTGCTTTGATAGTGAAATTAAGATGTCAGATG
Psteckii        TTGAATCATCGCAGACAAGCTTGTTGATCTGTTTTGATAGTGAAATGAGGATGTCGGATG
                ***.***.**********.***.******** ***** ******** *..*****.** *

Pcitrinum       TGATATCTTCACTTCCAACATTTGCTGTCTGAACACCCGCTACATTTGCAACGTCGAGCA
Psizovae        TGATATCTTCACTTCCAACATTTGCTGTCTGGACACCCGCGACATTTGCAATATCGAGCA
Psteckii        TGATATCTTCACTTCCCACATTCGCTGTCTGGACACCCGCTACATTTGCAATATCGAGCA
                ****************.***** ********.******** ********** .*******

Pcitrinum       TAGATGGAACAGCATCGATTAGAGCACGAACGCCAACCGCTCCAGAAGTGTAGCCTGTTG
Psizovae        TGGATGGAACGGCATCGATTAGAGCACGAACGCCAACCGCTCCAGAAGTGTAGCCTGTCG
Psteckii        TGGATGGGACGGCATCAATTAGAGCACGAACTCCAACCGCTCCAGAGGTGTAGCCTGTCG
                *.*****.**.*****.************** **************.*********** *

Pcitrinum       TTGCCGTTGAGCTTGAATCTGAGCCTGCAATTGTCCCTCCTAAGAGGTGTTAGGAAAATA
Psizovae        TTGCCGTTGAGCTTGAATCTGAGCCTGCAATTGTTCCTCCTAGGGAGTTTTAGATAAAAA
Psteckii        TGGCCGTCGAGCTTGAATCTGAGCCTGCTATAGTTCCTCCTATAAGGTGTCAGTAAAATA
                * ***** ********************:**:** ******* ...** * ** :***:*
```

```
                                     RASP6
Pcitrinum       TTAGCATTTGAACGCAGAATTGGATATAGGGGGCCAAACGAACCTGTAGCAAAGATGGTT
Psizovae        TTAGGATAGAAACGTG------GGATAAAGGAGTCGGACTGACCTGTAGCAAAAATGGTT
Psteckii        CTAGCATAATTACATG------GGAGAACCGGGTTGGACTAACCTGTAGCAAAAATGGTG
                 *** **:  :**. .      *.: :*  *.*  ..** .************.*****

Pcitrinum       ACATTAGGAAGGGTATGATTCATCTGAGTGAAATTCAACCCATTCGCATTGGTGAAAACA
Psizovae        ATATTAGGAAGGGTATGATTCATCTGGGTGAAATTCAGACCATTCGCATTGGTGAAAACA
Psteckii        ATATTAGGAAGGGTATGATTCATCTGGGTGAAATTCAACCCATTCGCATTGGTGAAAACG
                * ************************.**********..*******************.

Pcitrinum       AACCCTGTGCCAATGGCTCCACGGCCATATAGCAAGGGCGATGCCGAACTCTGACAAGCA
Psizovae        AACCCTGTGCCATTGGTTCCACGGCCATACAGCAGGGGCGAAGCCGAACTCTGACAAGCA
Psteckii        AACCCTGTACCATTGGCGCCACGACTATACAGCAGGGGCGAAGCCGAGCTCTGCCAAGCA
                ********.***:***  *****.* *** ****.******:*****.*****.******

Pcitrinum       AGAGTGGCCAACGTTATCAGGAAGCTCTTTATTGACACCATTGTGGAAATGCTTGGTCAG
Psizovae        AAAGTAGCCAACGCTACGAGGAAGCTCTTAATTGACACCATCGTGAAAAGGCCTGATCAG
Psteckii        AGAGTAGCCAACGCTACGAGGAAGCTTTTAATTGGCACCATTGTGGAAAGGCTTGCTTGG
                *.***.******* **  ******** **:****.****** ***.*** ** ** *  .*

Pcitrinum       ACTTGGGGTTCAACCTTAGAGACAATTTAATCCATGTCAACGATTTATGCCATTCTTAAG
Psizovae        ACTGGGG---------TTCAGCCAATTCAATCCATGTCAACGATT---------------
Psteckii        ACTTGGTACTCAACCTTAGAATCAATTCAATCAATGGCAACGATTTATGCCATTCTTAAA
                *** **           *: *. ***** ****.*** ********

Pcitrinum       GAA-TTTGTGATCTTATCGTACTACTTCATACATCCGCGATATACCACTAATTATCGGTT
Psizovae        ------------------------------------------------------------
Psteckii        GAAATCCGTGATCTTATCGTACTGCTTCATACTTCCGTGGAGTAC-AGTAATTATCGGTA
                 *** *  **************** ******** **** *    *** * ***********
                           R2
Pcitrinum       TGCCTATTACGTGGAATTGTGAATCTACTAG
Psizovae        -------------------------------
Psteckii        TGTCTATTACGTGGAATTGTGAATCTTCTAG
                ** ********************** ****
```

Figure S1. Nucleotide alignment of *P. citrinum* and *P. steckii* sequences to construct the L-asparaginase gene sequence of *P. sizovae* with the degenerate primers used in identification (reverse complement). Forward primers F1.2, F2, FASP2 and FASP3 (blue); reverse primers RASP2.1, RASP6 and R2 (pink); initiation codon (green); stop codon (red).

5’AATCGTTGACATGGATTGAATTGGCTGAACCCCAGTCTGATCAGGCCTTTTCACG**ATGGTGT
CAATTAAGAGCTTCCTCGTAGCGTTGGCTACTTTTGCTTGTCAGAGTTCGGCTTCGCCCCTGCT
GTATGGCCGTGGAACCAATGGCACAGGGTTTGTTTTCACCAATGCGAATGGTCTGAATTTCACC
CAGATGAATCATACCCTTCCTAATATAACCATTTTTGCTACAGGTCAGTCCGACTCCTTTATCC
CACGTTTCTATCCTAATTTTTATCTAAAACTCCCTAGGAGGAACAATTGCAGGCTCAGATTCAA
GCTCAACGGCAACGACAGGCTACACTTCTGGAGCGGTTGGCGTTCGTGCTCTAATCGATGCCGT
TCCATCCATGCTCGATATTGCAAATGTCGCGGGTGTCCAGACAGCAAATGTTGGAAGTGAAGAT
ATCACATCTGACATCTTAATTTCACTATCAAAGCAGATCAACAAGTTTGTCTGCGACGATCCAA
CCATGGCTGGGGCTGTTGTTACTCATGGAACCGATACTTTGGAGGAAACTGCTTTTTTCCTTGA
CGCTACGATCAATTGTGGTAAACCCGTCATCATTGTTGGCGCCATGCGTCCTTCAACCGCCATC
TCAGCTGATGGACCCTTCAATCTCCTTGAATCCGTTACCGTCGCAGCATCTCCGAAGGCTAAGA
ACCGCGGTGCCATGATTGTCATGAATGATCGCATCGCATCCGCTTACTACACGACCAAGACCAA
CGCCAACACCATGGATACGTTCAAAGCAATGGAAATGGGATACTTAGGAGAAATGATCTCTAAT
ACCCCATTCTTTTTCTACCCCCCCGTACAGCCGACGGGAAAGAAGGACTTCAACATTGCCAACG
TCACGGAAATTCCGAGAGTTGATATTCTCTTCTCTTATGAGGATATGCATAATGACACCTTGTA
CAATGCCATCGAAAGTGGCGCAAAGGGCATCGTGGTAGGTCATCCTTTCAGAATTGACAGAAAA
ATATTTCCCGTAAATGTTGTTTAACAGCTGACTGGTCGTAGATTGCAGGGGCTGGGGCCGGAGG**

AGTCACAACGTCATTCAACTATGCCATTGAAGACGCTATCAATCGCCTCGGAATCCCAATAATT
CAAAGTATGCGTACGGTCAATGGTGAAGTGCCATTGTCAGACGTCGAAAGCACCAGTGCCACTC
ATATTGCCAGTGGATACTTGAACCCTCAAAAATCCCGTATATTACTGGGACTATTGCTAGCGAA
GTCGAGCAACATCACTGAGATTGCTTCAACCTTTTCTCTTAATACAAATGCCTAGACTAGTGTT
TGGCAGAGAAGATTGCAAAAACCGATGACATATAGGCCACTCTTTGACTTGACCCCTACATACC
CACGTAGGTAGTTACAAAAACATTGATACTAGCAGTAAATAGAGAGTAACTTCGCAGAACATTA
TTTATTGATCTTTCCTTCCCCTATTCCCCATGTCATAATGGAGAAAGACGTACTAGTACAGTAT
AATTTGAGACAGACAGGGAGATTGCAGAACCAGTAGAATTATCCAACTTAAATGACATAAAAAC
TTTGCACCAGTATAATCTCGCTACACATAACGAACATCTGCAGATCGTCCCAATCTATTTCTTC
TCCCAACCGCCTTCGACATTCCCTTCGTCCGCGATATGATTGTATGGCTTATCGTACCGATTGT
CCATATCTTTATTGTGCTCCTTTACATCCTTAGGCAGGTCCTTACGG3’

Figure S2. *Penicillium sizovae* gene sequence. L-asparaginase (bold) and introns (underlined).

MVSIKSFLVALATFACQSSA**SPLLYGRGTNGTGFVFTNANGLNFTQMNHTLP**
NITIFATGGTIAGSDSSSTATTGYTSGAVGVRALIDAVPSMLDIANVAGVQT
ANVGSEDITSDILISLSKQINKFVCDDPTMAGAVVTHGTDTLEETAFFLDATI
NCGKPVIIVGAMRPSTAISADGPFNLLESVTVAASPKAKNRGAMIVMNDRI
ASAYYTTKTNANTMDTFKAMEMGYLGEMISNTPFFFYPPVQPTGKKDFNI
ANVTEIPRVDILFSYEDMHNDTLYNAIESGAKGIVIAGAGAGGVTTSFNYAI
EDAINRLGIPIIQSMRTVNGEVPLSDVESTSATHIASGYLNPQKSRILLGLLL
AKSSNITEIASTFSLNTNA-

Figure S3. Predicted amino acid sequence of the native and partial (bold) L-asparaginase gene from *Penicillium sizovae*.

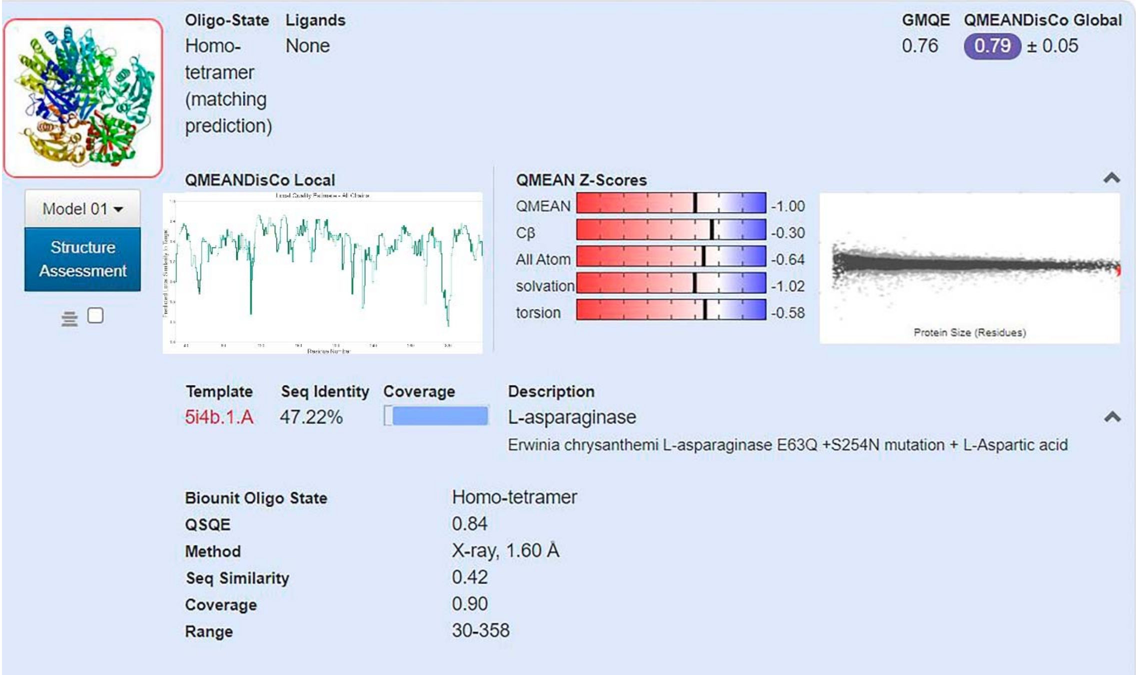

Figure S4. L-asparaginase crystal structure using Swiss model. Homology model prediction quality evaluation. QMQE: Global Model Quality Estimate (range 0-1); QMEANDisCo Global [60]: Model Quality Estimation with Distance Constraints.

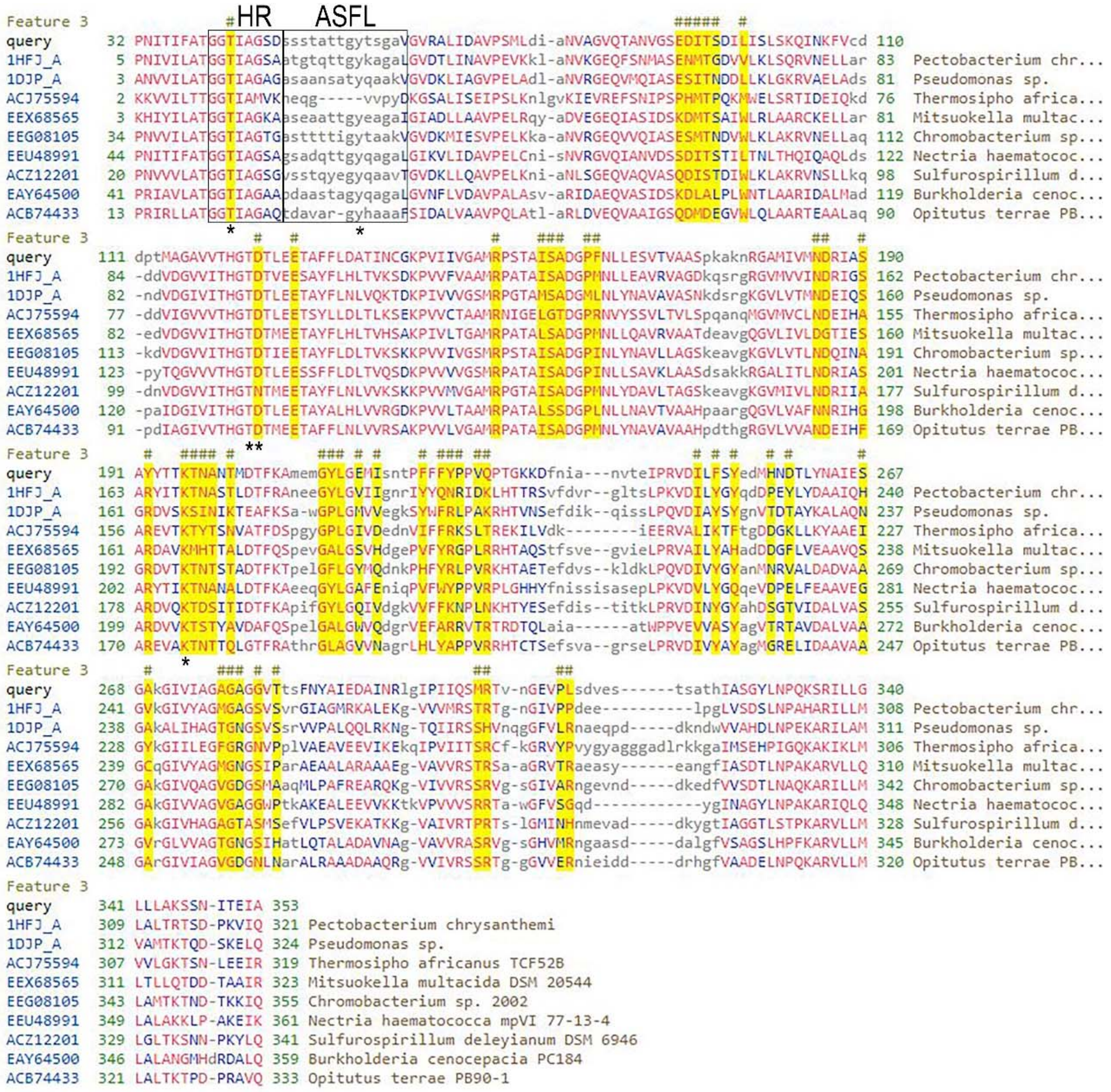

Figure S5. Alignment of L-asparaginases Conserved Protein Domain Family of type II L-asparaginases with the enzyme from *P. sizovae* (query). In yellow are the conserved residues of homotetrameric interfaces. HR: Hinge Region, ASFL: Active Site Flexible Loop. * Residues that form the active site.
